# Sensitivity and Specificity of a New Vertical Flow Rapid Diagnostic Test for the Serodiagnosis of Human Leptospirosis

**DOI:** 10.1371/journal.pntd.0002289

**Published:** 2013-06-27

**Authors:** Cyrille Goarant, Pascale Bourhy, Eric D'Ortenzio, Sylvie Dartevelle, Carine Mauron, Marie-Estelle Soupé-Gilbert, Lilian Bruyère-Ostells, Ann-Claire Gourinat, Mathieu Picardeau, Faridabano Nato, Suzanne Chanteau

**Affiliations:** 1 Institut Pasteur de Nouvelle-Calédonie, Association Pasteur International Network, Nouméa, New Caledonia; 2 Institut Pasteur, Biology of Spirochetes Unit, National Reference Center and WHO Collaborating Center for Leptospirosis, Paris, France; 3 Institut Pasteur, Plateforme 5, Paris, France; Institute of Tropical Medicine, Belgium

## Abstract

***Background***: Leptospirosis is a growing public health concern in many tropical and subtropical countries. However, its diagnosis is difficult because of non-specific symptoms and concurrent other endemic febrile diseases. In many regions, the laboratory diagnosis is not available due to a lack of preparedness and simple diagnostic assay or difficult access to reference laboratories. Yet, an early antibiotic treatment is decisive to the outcome. The need for Rapid Diagnostic Tests (RDTs) for bedside diagnosis of leptospirosis has been recognized. We developed a vertical flow immunochromatography strip RDT detecting anti-*Leptospira* human IgM and evaluated it in patients from New Caledonia, France, and French West Indies.

***Methodology/Principal Findings***: Whole killed *Leptospira fainei* cells were used as antigen for the test line and purified human IgM as the control line. The mobile phase was made of gold particles conjugated with goat anti-human IgM. Standards for Reporting of Diagnostic Accuracy criteria were used to assess the performance of this RDT. The Microscopic Agglutination Test (MAT) was used as the gold standard with a cut-off titer of ≥400. The sensitivity was 89.8% and the specificity 93.7%. Positive and negative Likelihood Ratios of 14.18 and 0.108 respectively, and a Diagnostic Odds Ratio of 130.737 confirmed its usefulness. This RDT had satisfactory reproducibility, repeatability, thermal tolerance and shelf-life. The comparison with MAT evidenced the earliness of the RDT to detect seroconversion. When compared with other RDT, the Vertical Flow RDT developed displayed good diagnostic performances.

**Conclusions/Significance:**

This RDT might be used as a point of care diagnostic tool in limited resources countries. An evaluation in field conditions and in other epidemiological contexts should be considered to assess its validity over a wider range of serogroups or when facing different endemic pathogens. It might prove useful in endemic contexts or outbreak situations.

## Author Summary

The major burden of leptospirosis happens in low-income populations from tropical or subtropical regions. Because of nonspecific symptoms in human leptospirosis, the biological confirmation is needed to ascertain the disease. However, this biological diagnosis relies on sophisticated and time-consuming techniques that are most often hardly (if ever) available to clinicians in peripheral health centers. Yet, the outcome of leptospirosis in humans largely depends on an early antibiotic treatment. Taken together, these factors highlight the need of rapid simple diagnostic tests for leptospirosis that could be used directly on the bedside even in remote health centers. In this study, we developed and evaluated a prototype point of care strip test for the serological diagnosis of human leptospirosis in New Caledonia, mainland France, and the French West Indies. The sensitivity was 89.8% [95% CI, 84.7–93.4] and the specificity 93.7% [95% CI, 89.65–96.2]. This easy, early and portable diagnostic test will be evaluated in other epidemiological conditions and under field conditions.

## Introduction

Leptospirosis is a bacterial disease of high incidence in many tropical and sub-tropical areas [1], [2]. Its clinical presentation is both highly variable and most often characterized by non-specific signs and symptoms; the complete triad of Weil's disease (hepatic failure, renal failure and hemorrhage) is recognized to account for less than one third of human cases [3], [4]. Most of the early signs and symptoms point to the so-called “acute fever of unknown origin” (FUO), a major diagnostic challenge in tropical and subtropical areas. Beside the epidemiological context and patient exposure history, to quickly diagnose and implement an appropriate treatment, an etiological investigation is necessary, especially in malaria, hantavirus, or viral hepatitis endemic regions or during influenza, chikungunya or dengue outbreaks. Leptospirosis is also reported to be an emerging or re-emerging disease in industrialized countries, with probable increasing impacts due to global warming and increasing travel-related cases [5], [6]. Unfortunately, the biological confirmation of leptospirosis is both tedious and rarely available in a timely manner. It notably requires sophisticated techniques that are most frequently available only in central reference laboratories [1]. These techniques are of prime importance in disease surveillance and epidemiological investigations but are inappropriate for early clinical care in peripheral health centers that support a major part of the leptospirosis burden. Additionally, an early and proper antibiotic treatment is known to be a key to rapid recovery and a major determinant of outcome in leptospirosis [3], [7], [8]. The need for portable rapid diagnostic tests (RDT) is striking and largely recognized to improve clinical management of leptospirosis patients, notably in remote dispensaries of tropical and subtropical regions. As an example, the major part of the leptospirosis burden in New Caledonia occurs away from the city and the central hospital of Noumea [9], [10].

In this study, we developed a vertical flow immunochromatographic RDT for the early diagnosis of leptospirosis to detect Anti-*Leptospira* human IgM and evaluated its sensitivity, specificity, reproducibility, repeatability, temperature stability and simulated shelf-life in the context of leptospirosis endemic (New-Caledonia and French West Indies) or non-endemic (mainland France) countries using clearly defined case definitions and the reference Microscopic Agglutination Test (MAT) results as the gold standard [4].

## Methods

### Antigen preparation

The antigen was prepared at the Institut Pasteur, National Reference Center for Leptospirosis (Paris, France) as follow: a culture of *L. fainei* serovar Hurstbridge strain BUT 6^T^ at OD = 0.5 at 420 nm was killed by 0.2% formalin for 3 hours at ambient temperature, boiled for 45 minutes and its pH adjusted to 9.6. This preparation was kept at 4°C until use as an antigen for the Vertical Flow RDT and was also used for an in-house IgM ELISA at the National Reference Center [11](Bourhy et al., manuscript in revision).

### Development and production of RDTs

The positive control line was made of purified human IgM (MP Biomedicals) at 2 mg/mL. Both control and test lines were sprayed as lines onto nitrocellulose membranes. Gold particles labelled with goat anti-human IgM (BBI International BA.GAHM40/X) adjusted to the concentration of OD_520 nm_ = 3 were used as the capture mobile phase to construct our one-step vertical flow immunochromatography RDT, as previously described [12].

Two batches of RDT were produced at the Institut Pasteur in Paris (platform 5) and used in this study: batch numbers 110,211 and 120,511.

### Case definitions and gold standard

Leptospirosis cases were defined as confirmed when a clinical and epidemiological suspicion was complemented by either a positive specific PCR evidencing genomes of pathogenic *Leptospira* spp. in the blood or urine of the patient, or when the MAT on acute and convalescent sera showed a seroconversion (from nil to a titer ≥400) or a significant seroascencion (at least a fourfold raise in titers) [13]. Because most of the serum specimens originated from leptospirosis endemic regions where a high MAT titer threshold is usually used, we adopted this ≥400 threshold throughout the study. Probable cases were defined as clinical and epidemiological suspicion together with a unique serum with a MAT titer ≥400. The panels of strains used for MAT were adapted to the local epidemiology of New Caledonia, mainland France and French West Indies [9], [14] and are provided in details in Table S1. High rates of agglutination of the serum with one particular strain are used to identify the presumptive serogroup of the infecting bacterium as described elsewhere [4].

### Study area and selection of sera for the evaluation

All sera used in this study were addressed to the Institut Pasteur in Nouméa or Paris for diagnostic purpose and originated from patients from New Caledonia, mainland France or the French West Indies (Martinique and Guadeloupe). The workflow is summarized as a flowchart in Figure S1. These laboratories are territorial (New Caledonia) and national (France and West Indies) reference centers for leptospirosis and receive all (New Caledonia) or more than 75% (France and French West Indies) of leptospirosis diagnosis requests. Sera were stored at −20°C, selected according to case definitions, and then tested blindly with RDTs. To assess the sensitivity of the RDT, only MAT-positive sera from confirmed cases were used: we tested 187 confirmed leptospirosis cases sera with a MAT titer ≥400 (120 from New-Caledonia, 38 from mainland France, 29 from French West Indies). The specificity was assessed using 221 sera (142 from New-Caledonia, 79 from mainland France): 12 anti-Chikungunya virus IgM positive sera, 58 anti-dengue virus IgM positive sera from all 4 serotypes, 6 anti-hepatitis A virus total Ig positive sera, 7 rheumatoid factor positive sera, 25 syphilis (TPHA and VDRL) positive sera, one acute malaria serum and 112 sera from healthy blood donors (Platform ICAReB, Institut Pasteur). These 221 sera were then confirmed to be MAT-negative (titer<100) within the same week.

### Evaluation of the test

Preliminary experiments determined dilution of sera between 1/100 and 1/800 in Phosphate Buffer Saline (PBS, pH 7.4) as suitable for testing with RDT. Because rapid tests are mostly to be used in endemic regions and similarly to the high MAT titer threshold, a 1/400 dilution of sera was used throughout the study. Briefly, Vertical Flow RDT strips were introduced into 200 µL diluted serum in 5 mL polystyrene tubes, for 15 minutes. The strips were then removed and placed on absorbent paper and read within 5 minutes. All results were recorded using a grading scale from 0 (no visible trace on test band) to 3+ (intensity of the test band equal to the intensity of the control band). The grading included a “weak” value for low but visible traces on the test band. Weak, 1+, 2+ and 3+ were then considered positive for further analysis and 0 was considered as negative.

### Sensitivity and specificity

All analyses were run blindly: any person involved in one particular analysis had no access to the results of the other tests results from the same serum.

To assess the sensitivity of the RDT, only the first MAT-positive serum from each confirmed case was used. For specificity evaluation, all 221 negative control sera have been tested using MAT and were all negative (titer<100) (see Table 1).

**Table 1:** Performance of the IgM RDT assay on laboratory-confirmed leptospirosis and controls.

	Samples	Number of sera	Number of positive sera (%)
Leptospirosis patients	New Caledonia	120	102 (85.0)
	French West Indies	29	29 (100.0)
	Mainland France	38	37 (97.4)
	Total	187	168 (89.8)
Controls	Blood donor controls	112	9 (8.0)
	Dengue patients	58	5 (8.6)
	Syphilis patients	25	0 (0)
	Others	26	0 (0)
	Total	221	14 (6.3)

Possible false negative results due to high levels of anti-Leptospira IgM (prozone phenomenon) was controlled using two positive sera with 25,600 and 51,200 MAT titers: serial two-fold dilutions of the sera (1/400 to 1/6,400) were tested and test band intensity of the Vertical Flow RDT were compared.

### Temperature stability and accelerated aging method for shelf life

The strips were stored at 4°C in sealed aluminium foils with a silica gel bag to avoid exposition to humid conditions, and the test was performed at laboratory room temperature (20°–25°C in New Caledonia and Paris).

The predictive shelf life of the RDT was assessed by testing serial dilutions of a MAT-positive serum (MAT titer = 800, pointing to serogroup Icterohaemorrhagiae) twice per week over a period of 3 weeks exposure of the strips at 60°C. During this period, the positive control serum was kept at 4°C to avoid repeated freeze-thaw cycles. This accelerated stability method is equivalent to two years of actual storage time at 25°C [15], [16].

### Reproducibility and repeatability

Several experiments were performed in New Caledonia to evaluate the the Vertical Flow RDT. For reproducibility and repeatability, four sera (3 positives and one negative) were tested blindly by three different operators on three different days; one serum was tested blindly 14 times by integrating 14 aliquots of the same specimen within 4 independent experiments and using two different batches of RDTs by the same operator; 177 sera from both confirmed and probable cases (28 negative and 149 positive samples) were read independently by two technicians, including 157 sera (16 negative and 141 positive samples) by three technicians. We additionally simulated tropical field conditions by performing the tests in parallel at 37°C in an incubator (simulating tropical conditions) and at laboratory temperature (standard condition)

### Comparative kinetics of seroconversion using MAT and RDT

The earliness of IgM seroconversion using MAT or RDT was assessed on serial sera (day 2 to day 18 after the onset of symptoms) from 17 confirmed cases, based on the date of onset as declared by the patients.

The RDT was also tested on early sera from 99 patients who were tested positive by PCR but negative by MAT (titer = 0, 100 or 200).

### Concordance between RDT and MAT on unique MAT positive sera (probable cases of leptospirosis)

One hundred and fifty MAT-positive sera from probable cases, including 124 sera from the IPNC collection and 26 from the French National Reference Centre, were tested using RDT.

### Comparison with three commercial diagnostic assays

To compare the newly developed RDT with currently available techniques, we compared its performance on identical sera from New Caledonia. To assess the sensitivity, 72 MAT-positive sera from confirmed cases were randomly selected from the 118 New Caledonian control sera. For the specificity, 72 negative controls were randomly-selected, corresponding to 10 anti-Chikungunya virus IgM positive sera, 30 healthy blood donors, 11 anti-dengue virus IgM positive sera from all 4 serotypes, 6 anti-hepatitis A virus total Ig positive sera, 7 rheumatoid factor positive sera, 7 syphilis (TPHA and VDRL) positive sera, one acute malaria serum. The results using our RDT were compared with those obtained using two Elisa assays (Leptospira IgM ELISA, Panbio, Inverness Medical, QLD Australia, and SERION ELISA classic Leptospira IgG/IgM, Institut Virion/Serion GmbH, Germany) and one lateral flow IgM immunochromatography assay (Leptocheck, Zephyr Biomedicals, India). The Serion ELISA test was used together with the Rheumatoid Factor Absorbent as recommended by the manufacturer. All tests were made within a 5 day period. For calculations, the “uncertain” results of ELISA were considered as positive.

### Statistical analysis

The evaluation of our RDT for the serodiagnosis of leptospirosis was performed according to the WHO Tropical Diseases Research Diagnostics Evaluation Expert Panel for the evaluation of diagnostic tests for infectious diseases [17].

Data were captured into Excel 2007 (Microsoft Corporation, Redmond, United States of America).

We calculated sensitivity (Se), specificity (Sp), positive and negative predictive values (PPV and NPV, respectively) of the RDT, using the reference MAT serology as the gold standard. The 95% confidence intervals (95% CI) were calculated using the Wilson's method. The variations of the PPV and NPV according to the prevalence of the disease were also plotted.

We also calculated likelihood ratios (LR). The positive LR (LR+ = Se/[1 - Sp]) indicates how many times a positive result is more likely to be observed in specimens with the target disorder than in those without the target disorder. The negative LR (LR− = [1 - Se]/Sp) indicates how many times a negative result is more likely to be observed in specimens with the target disorder than in those without the target disorder. The more accurate the test is, the more LR differs from 1. LR+ above 10 and LR− below 0.1 are considered convincing diagnostic evidence [18]. The 95% CIs were calculated for LR+ and LR− [19].

The diagnostic odds ratio (DOR) measures the test performance by combining the strengths of sensitivity and specificity, with the advantage of representing a single indicator of accuracy. These characteristics make the DOR particularly useful for comparing tests whenever the balance between false negative and false positive rates is not of immediate importance [20]. The DOR is defined as the ratio of the odds of positive test results in specimens with the target disorder relative to the odds of positive test results in specimens without the target disorder. It was calculated as follows:
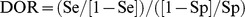
The DOR does not depend on prevalence and its value ranges from 0 to infinity, with higher values indicating better discriminatory test performance. The 95% CIs for DOR values were also calculated [21].

### Samples, ethics statement

The IPNC and the French NRC are reference diagnostic laboratories for leptospirosis. In New Caledonia, leptospirosis is a notifiable disease. The serum samples used in this study were selected from the 2008–2001 IPNC and 2009–2011 French NRC collections of sera issued from routine diagnostic activities and as part of public health surveillance. This biobank of sera was declared to the French Ministry of Research (DC-2010-1222, Collections number 1 and 2). This study was part of a protocol approved by the Institut Pasteur (protocol # RBM2008-16) and the French Ministry for Education & Research (protocol # AC-2007-44). All sera were tested as anonymous samples. Negative sera from mainland France were provided by Platform ICAReB (Investigation Clinique et Acces aux Ressources Biologiques). The STARDT checklist is provided as Table S2.

## Results

### Evaluation of the test

The sera used in this study were from patients or donors of both sex and all age classes, being selected among leptospirosis suspicions (positive sera), other pathologies or blood donors (negative sera). All along the study and whatever the batch used, we observed no invalid test: all RDT displayed an intensely marked control line and very little to no background coloration. Out of the 187 gold standard positive sera tested, 168 were RDT positive, including 15 RDT with a test line intensity graded as “weak” (8.9% of positives). The putative serogroups of the 19 RDT negative sera were: Icterohaemorrhagiae (n = 12), Pyrogenes (n = 3), Australis (n = 2), Panama (n = 1) and one could not be determined due to co-agglutination of multiple serogroups.

Out of the 221 MAT-negative sera tested, 207 were RDT negative. All 14 RDT positive sera were graded “weak” and originated from 9 healthy blood donors and five patients positive for anti-dengue virus IgM.

The sensitivity and specificity of the RDT were therefore, respectively, Se = 89.8% [95% CI, 84.7–93.4] and Sp = 93.7% [95% CI, 89.65–96.2]. The Likelihood Ratios (LR) were therefore LR+ = 14.18 [95% CI, 8.52–23.56] and LR− = 0.11 [95%; 0.01–0.17]; and the Diagnostic Odds Ratio DOR of 130.74 [95% CI, 63,65–268,52].

**Figure 1:**
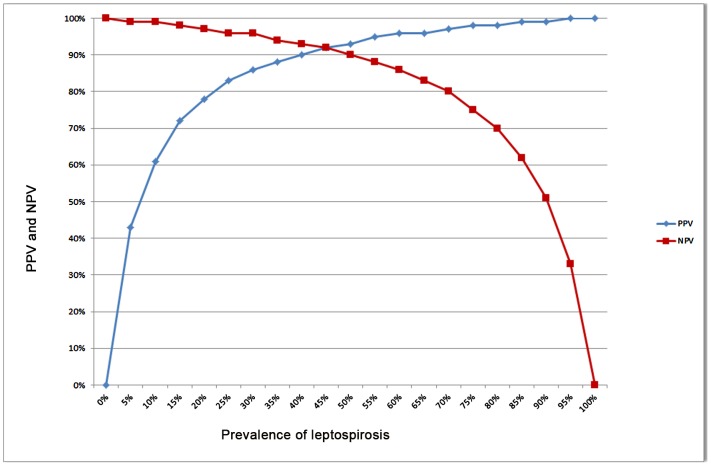
Positive and negative predictive values (PPV, NPV) of the Vertical Flow Rapid Diagnostic Test. Values calculated using 187 positive and 221 negative serum specimens.

The results are summarized in Table 1, positive and negative predictive values of our RDT according to prevalence are presented in Figure 1.

The absence of false negative due to prozone phenomenon was demonstrated using dilutions of sera with highest MAT titers: serial two-fold dilutions actually yielded test lines of decreasing intensities..

### Temperature stability and accelerated aging method for shelf life

To simulate tropical conditions, the RDT results of 10 MAT-positive sera run at 37°C were compared and proved identical to those run at 25°C. For accelerated shelf-life evaluation, serial two-fold dilutions (from 1/400 to 1/12,800) of one MAT positive serum (titer 800) were tested twice a week for three weeks with RDT stored at 60°C. At day 1, the RDT was positive at a 6,400 dilution, and remained the same until day 17. At day 21, the 3,200 dilution was the last giving a positive test line (a one dilution decrease of the serum).

### Reproducibility and repeatability

One serum tested 14 times with strips from the two different batches gave 14 similar results, including the grade of the test line.

Inter-readers variability was assessed by two independent operators on 177 sera (28 negative and 149 positive) of which 157 sera were read by three independent operators. These readings provided an excellent inter-operator agreement (>99%) in all cases but one: one weakly positive RDT from a probable case was rated “weak” by two operators but negative by the third one.

Inter-operator variability was also assessed using 4 sera (RDT graded from negative to 3+) blindly and independently tested on three different days by three different operators. Two operators provided perfectly concordant grading results on all three tests, the third one graded “weak” a negative serum once out of the three tests.

### Comparative kinetics of MAT and RDT

Of 17 confirmed cases analysed (see Table 2), one patient (number 1) seroconverted for MAT at day 6 (pointing to Icterohaemorrhagiae) but remained negative for RDT; oppositely, 5 PCR-confirmed patients (numbers 2–6) were MAT negative whereas they were RDT-positive. For one of these patients (number 6), PCR and RDT tests were both positive at day 4 after onset of symptoms. Five patients (numbers 7–11) were positive for MAT and RDT on the same day (days 5–11 after the onset of symptoms); lastly, for 6 patients (numbers 12–17), the RDT was positive earlier than the MAT (day 3 to day 7). Out of these 6, four (numbers 12, 15, 16 and 17) had a positive blood PCR and RDT results on the same day (on days 5, 4, 7 and 3 respectively).

**Table 2:** Comparative kinetics of antibody detection using MAT and RDT.

Patient	Leptospirosis diagnosis	Putative infecting serogroup	Sera available and tested (Day(s) since onset of symptoms)*	MAT≥400 (Day(s) since onset of symptoms)*	RDT positive (Day(s) since onset of symptoms)*
1	blood PCR+ at D4	Icterohaemorrhagiae	D4-6	at D6	negative
2	blood PCR+ at D8	Icterohaemorrhagiae	D8-9; D11-14	negative	D9
3	blood PCR+ at D2	Icterohaemorrhagiae	D2-6	negative	D5
4	blood PCR+ at D4	Icterohaemorrhagiae	D4; D6-12	negative	D6
5	blood PCR+ at D1	Pyrogenes	D1-2; D4-5; D7	negative	D7
6	blood PCR+ at D4	Ballum	D4-6	negative	D4
7	blood PCR+ at D6	Icterohaemorrhagiae	D6-8; D11	at D11	at D11
8	blood PCR+ at D4	Icterohaemorrhagiae	D4-8	from D7 on	from D7 on
9	Seroconversion D4-D7	Icterohaemorrhagiae	D4; D7; D9	at D7 and D9	at D7 and D9
10	urine PCR+ at D5	Icterohaemorrhagiae	D5-6	at D5 and D6	at D5 and D6
11	Seroascension D5-D9	Icterohaemorrhagiae	D6, D9, D11	from D6 on	from D6 on
12	blood PCR+ at D5	Icterohaemorrhagiae	D5-8; D10; D12-13	at D13	from D5 on
13	urine PCR+ at D8	Icterohaemorrhagiae	D7-12; D17	at D17	from D7 on
14	blood PCR+ at D3	Icterohaemorrhagiae	D3-6	at D6	from D5 on
15	blood PCR+ at D4	Icterohaemorrhagiae	D3-7; D9-12	from D5 on	from D4 on
16	blood PCR+ at D7	Icterohaemorrhagiae	D7-11; D13-17	at D17	from D7 on
17	blood PCR+ at D3	Icterohaemorrhagiae	D3-6	at D6	from D3 on

* D0 is the day of onset of symptoms as declared by the patient.

Similarly, in 16 out of 99 early sera from confirmed patients from New Caledonia, the RDT was positive whereas the MAT was still negative (6 out of 62 MAT negative) or displayed low titers (4 out of 21 with a MAT titer of 100; and 6 out of 16 with a MAT titer of 200).

### Concordance between RDT and MAT in patients diagnosed as “probable cases”

Of 150 sera from probable cases of leptospirosis (unique sera with a MAT≥400), 109 gave a positive result using the RDT, corresponding to a concordance of 72.7% [65–79.1].

Out of these, 108 had a MAT>400, from which 81 (75% [66.1–82.2]) were RDT-positive, while 63 had a MAT titer >800, from which 53 (84.1% [73.2–91.1]) were RDT- positive.

### Comparison of our RDT with three commercial diagnostic assays

**Figure 2:**
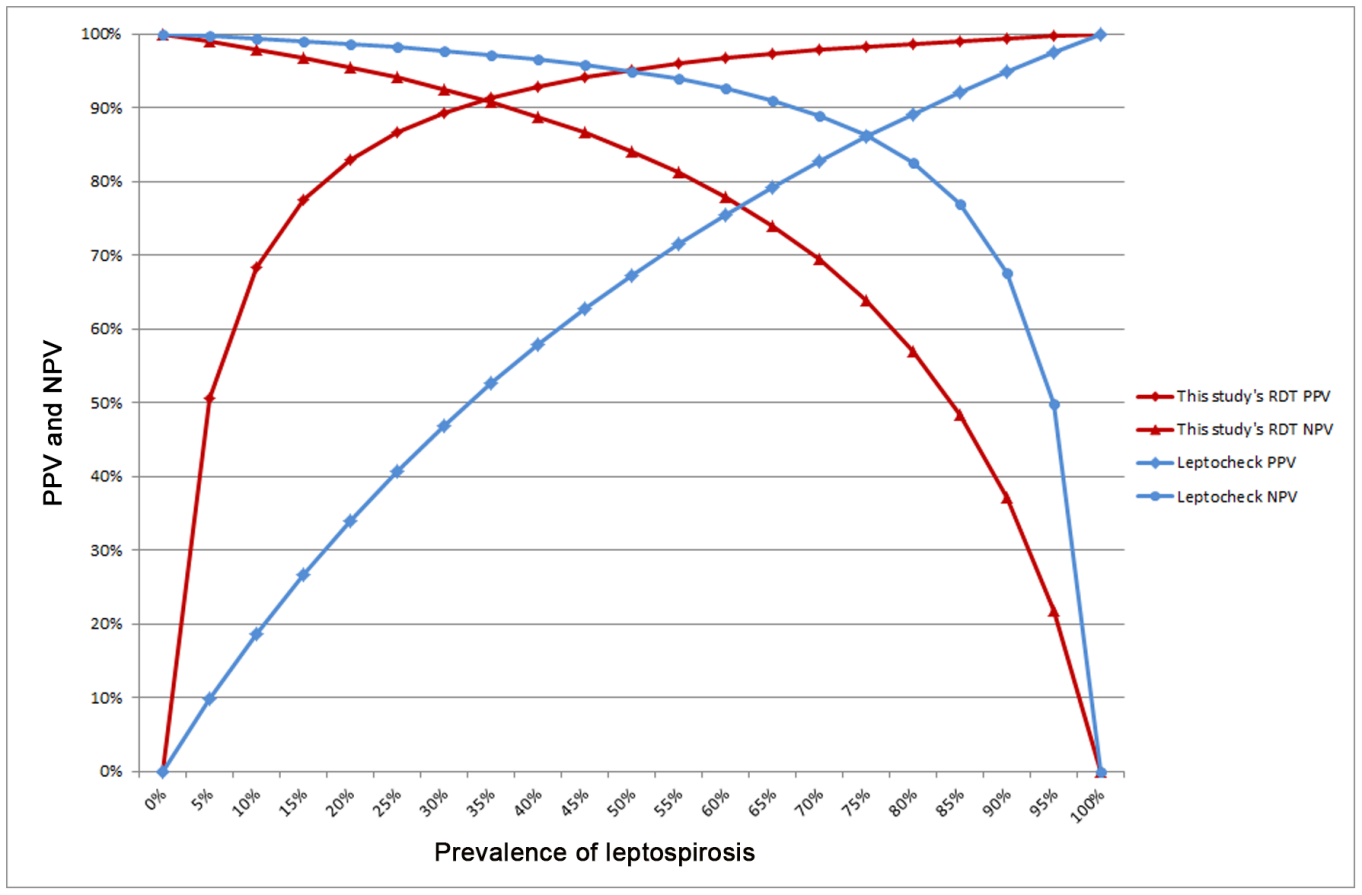
Comparative predictive values of the Rapid Diagnostic Test and of a commercial test. Positive and negative predictive values for the diagnosis of leptospirosis using the IgM Rapid Diagnostic Test evaluated in this study and a commercial lateral flow IgM assay were calculated using 72 positive and 72 negative serum specimens randomly selected from New Caledonian specimens.

The use of 72 gold standard positive (MAT≥400 from confirmed cases) and 72 negative (MAT<100) serum specimens selected randomly allowed a comparison of our RDT with three commercially available tests: two ELISA tests (from Serion (using the recommended RF absorbant) and from Panbio) and one IgM lateral flow immunochromatographic assay (Leptocheck). The results of these tests are detailed in Table 3. The IgM ELISA from Panbio had 100% specificity on these particular specimens together with the lowest sensitivity (75%). This 100% specificity does not allow the calculation of a Diagnostic Odds Ratio (DOR) that would however be very high. The ELISA test from Serion had both a good sensitivity (91.7%) and a good specificity (81.9%), therefore showing a good DOR of 49.9. Another rapid diagnostic test, namely Leptocheck (from Zephyr) had a very good sensitivity (91.2%) but a quite low specificity (52.8%), giving a DOR of 39.1. The Vertical Flow RDT we developed displayed a very good specificity (95.8%) and a good sensitivity (81.9%) and had therefore a very good DOR of 104.4. The corresponding curves of predictive values according to the prevalence of the two IgM rapid tests on these specimens are compared in the figure 2.

**Table 3:** Comparison of four rapid diagnostic tests on randomly-selected serum samples.

MAT	Pasteur RDT 1/400	Leptocheck	Elisa Serion (+RF absorbant)	Elisa Panbio
Positive 72	Positive 59	Positive 59	Positive 58	Positive 53
				Negative 5
			Negative 1	Positive 0
				Negative 1
		Negative 0	Positive 0	Positive 0
				Negative 0
			Negative 0	Positive 0
				Negative 0
	Negative 13	Positive 11	Positive 8	Positive 1
				Negative 7
			Negative 3	Positive 0
				Negative 3
		Negative 2	Positive 0	Positive 0
				Negative 0
			Negative 2	Positive 0
				Negative 2
**Sensitivity**	**81.9% [71.5–89.1]**	**97.2% [90.4–99.2]**	**91.7% [83–86.1]**	**75% [63.9–83.6]**
Negative 72	Negative 69	Negative 38	Negative 59	Negative 72
**Specificity**	**95.8% [88.4–98.6]**	**52.8% [41.4–63.9]**	**81.9% [71.5–89.1]**	**100% [94.9–100]**
**LR+** *	**19.7 [6.5–59.9]**	**2.1 [1.6–2.6]**	**5.1 [3.1–8.3]**	**NA**
**LR−** †	**0.2 [0.1–0.3]**	**0.05 [0.01–0.21]**	**0.1 [0.05–0.2]**	**0.25 [0.17–0.37]**
**DOR** ‡	**104.4 [28.4–384]**	**39.1 [8.9–171.8]**	**49.9 [17.8–139.7]**	**NA**

NA: Not Applicable.

* LR+: Positive Likelihood Ratio – [95% CI].

† LR−: Negative Likelihood Ratio – [95% CI].

‡ DOR: Diagnostic Odds Ratio – [95% CI].

Sensitivity (%), number of positive rapid diagnostic test among patients with serological evidence (MAT) of leptospirosis (n = 72) – [95% CI].

Specificity (%), negative rapid diagnostic test among serum samples from patients with no serological evidence (MAT) of leptospirosis (n = 72) – [95% CI].

## Discussion

Serum samples from leptospirosis patients contain antibodies that become detectable approximately one week after the onset of symptoms [4]. MAT is a long used gold-standard method for the serological diagnosis of leptospirosis. This method relies on the detection of agglutinating antibodies (both IgM and IgG) against antigens that are live *Leptospira* strains corresponding to representative serogroups of epidemiological significance. However, many laboratories and hospitals do not have the facilities and expertise required to perform the MAT. Therefore, there is a growing use of qPCR for early diagnosis [22], providing the opportunity to rapidly confirm leptospirosis suspicions in acute phase sera. More simple and rapid serological diagnostic tests including ELISA-based assays detecting antibodies are also used. However, none of these techniques are prone to be implemented in health centers of endemic regions where the highest burden of leptospirosis occurs.

Pathogenic *Leptospira* strains are classified into 9 species and more than 200 serovars reflecting the structural heterogeneity in the carbohydrate component of the lipopolysaccharide. ELISA-based assays using crude whole-cell lysates of *Leptospira* strain (usually the saprophyte *L. biflexa* serovar Patoc strain Patoc 1) as antigens may not recognize the diversity of circulating strains and the sensitivity of these tests are generally poor [3], [4]. A major challenge is to discover antigens that are conserved across the major leptospiral strains. In this study, we tested a one-step vertical flow immunochromatography RDT coated with heat-killed *L. fainei* serovar Hurstbridge as an antigen. *L. fainei* belongs to the intermediate group of *Leptospira* [23] and, as such, may share common antigenic features with saprophytes and pathogens which constitute the two other phylogenetic groups in the genus *Leptospira*. In addition, a previous study has suggested that *L. fainei* serovar Hurstbridge may cross-react with different pathogenic serovars [24].

Rapid diagnostic tests should ideally be accurate, simple to use, relatively inexpensive, easy to interpret, stable under extreme conditions, with little or no processing, and give the results within less than 2 hours.

A proper evaluation of a diagnostic test has to face two major challenges: first, the samples used for validation must have a very well-defined status with regard to the diagnostic target of interest; second, the results of the test under evaluation must be compared with the results of the same samples characterized using a validated reference test defined as the “gold standard”. Our study evaluated the sensitivity and specificity of a new Vertical Flow RDT for the serological diagnosis of leptospirosis in endemic (New Caledonia and French West Indies) and non-endemic (mainland France) countries. We only used sera from confirmed leptospirosis cases [13] for this evaluation. Therefore, the positive samples for the evaluation of sensitivity were both gold standard positive (a MAT titer of at least 400) and from confirmed leptospirosis cases (either a positive PCR or a seroconversion from nil to ≥400 or a ≥4-fold rise in MAT titer in paired sera). This high MAT threshold was chosen because most of the specimens (149 out of 187) originated from endemic regions, where a similar MAT threshold is used for diagnosis and surveillance [4], [9]. Additionally, negative sera for specificity evaluation were tested blindly using the reference MAT and were only considered as true negatives if the MAT titer was below 100. These latter originated from both healthy volunteers and a selection of patients with pathologic conditions of relevance in endemic countries. Using this clearly defined case definition, sensitivity and specificity were assessed using a high dilution of sera (1/400) reflecting the high MAT threshold titer. The sensitivity and specificity of the new Vertical Flow RDT in these conditions were 89.8% and 93.7% respectively. These results compare and are slightly better than the ones reported by Smits et al. who reported a 85.8% sensitivity and a 93.6% specificity with another Vertical Flow RDT [25].

The performance of ELISA tests vary widely in terms of sensitivity and specificity. For example, a commercial IgM ELISA (Panbio) gave a sensitivity and specificity of 76% and 82% in northeast Thailand [26], 35% and 98% in Hawai [27], and 61% and 66% in Laos [28], respectively. Reported variations in diagnostic assay performance may reflect population-related differences such as past exposure to leptospirosis or environmental leptospires. This can also be attributable to differences in the choice of the case definition; MAT is usually used as the reference test [29].

To increase the statistical power of our evaluation, we included as many serum samples as possible, only including the earliest positive serum when serial samples were available. Because the patient population recruited through our laboratories represents all (New Caledonia) or around 80% (France and West Indies) of leptospirosis suspicions, our collection can be regarded as representative of the total patient population in these regions. These included sera from New Caledonia older than 3 years, stored frozen at −20°C. It is well recognized that the long term storage of serum specimens at −20°C and their freeze/thawings may result in a drop of IgM titers. Actually, the sensitivity was higher in sera stored for less than two years than in sera stored for more than two years (90.6% versus 81.5%). This may have resulted in a slight under-estimation of the sensitivity of this Vertical Flow RDT.

The RDT we developed reacts with IgM to at least serogroups Australis, Autumnalis, Ballum, Bataviae, Canicola, Cynopteri, Grippotyphosa, Hebdomadis Icterohaemorrhagiae, Panama, Pomona, Pyrogenes, Sejroe and Tarassovi, indicating that the assay reacts broadly with antibodies mounted against *Leptospira* strains circulating worldwide.

Probable leptospirosis cases were defined as cases with a leptospirosis-compatible clinical presentation but a unique serology with a MAT titer ≥400. We tested our Vertical Flow RDT using these unique sera from such cases. Interestingly, the proportion of Vertical Flow RDT -positive sera was significantly lower than the sensitivity of the test as determined using confirmed cases (69.4% versus 89.8%, χ^2^ = 9.08, p<0.01), suggesting a poorer positive predictive value of the RDT for patients classified as probable cases. Two main reasons might contribute in explaining this difference. First, our Vertical Flow RDT only detects IgMs whereas the MAT is known to detect both IgMs and IgGs. Because IgM titers are known to decline faster than IgG, some positive MAT results may reveal IgGs remaining from previous exposure to leptospires. MAT could therefore be less specific than IgM-specific assays to detect acute and recent leptospirosis. It is also well known that direct visual methods such as MAT (agglutination of bacteria using microscopy) are less sensitive than indirect amplified techniques such as ELISA or colloidal gold particles immunochromatography assays. The possibility of false positive MAT results was already observed in other contexts [30]. Actually, though MAT is the recognized reference technique for the serological diagnosis of leptospirosis, it also suffers some drawbacks and weaknesses. Some concerns about both its sensitivity and its specificity have been raised and discussed [30]–[36]. More recently, a mathematical modeling study again demonstrated the limitations of MAT as a gold standard [29]. Since no diagnostic assay is adequately sensitive and specific enough to diagnose all acute cases of leptospirosis, results should be confirmed by another method. Regarding sensitivity, the most widely recognized weakness of MAT is its low sensitivity in early acute phase sera. The ability to detect anti-*Leptospira* antibodies earlier in the course of the disease with specific IgM detection tests than with MAT has already been largely recognized [30], [33], [34], [37]–[41]. We also observed an earlier positivity of our IgM Vertical Flow RDT when compared to MAT in serial sera. In our study, 11 out of 17 leptospirosis seroconversions could be diagnosed earlier with the Vertical Flow RDT than with the MAT (Table 2). Similarly, in 16 out of 99 early sera from confirmed cases, the Vertical Flow RDT was positive before the seroascension (10/37) or seroconversion (6/62) could be evidenced with the MAT. However, for strictness reasons, our strategy was to only use gold standard-positive specimens (MAT titers ≥400) for sensitivity assessment. Therefore, the sensitivity evaluated here might not reflect the conditions of rapid tests use in routine medical conditions, where patients may be seen and tested before seroconversion.

Previous studies have generally found that ELISA-based assays detect anti-Leptospira antibodies earlier in the course of the disease than with MAT [4], [30], [33], [37], [38]. Anti-leptospiral IgM cannot be detectable before 4–5 days after onset of symptoms, before the appearance of IgG and agglutinating antibodies [42].

Because an early diagnosis is of prime importance in the clinical management of leptospirosis [43], the possibility to ascertain the disease earlier in the course of the infection should be regarded as a real asset.

When considering the need of RDT for bedside diagnosis, the comparison of our Vertical Flow RDT with a RDT that is commercially available shows that our test has a lower sensitivity (81.9% versus 97.2%) but a much higher specificity (95.8% versus 52.8%) and therefore a better Diagnostic Odds Ratio (104.4 versus 39.1). This better performance is also shown by the comparison of the curves of their predictive values according to prevalence (Figure 2). There may be various reasons for these differences, including a different antigen used for IgM detection. However, the most probable cause is a much lower dilution of serum specimens in the Leptocheck lateral flow IgM assay (ca. 1/20) when compared with the dilution used for our Vertical Flow RDT (1/400). Because the MAT positive threshold titers may vary depending on the region [44], endemic countries usually using a higher threshold, it might be worth also considering using a different serum dilution for RDT according to the local epidemiology of leptospirosis. Because leptospirosis is endemic in New Caledonia and French West Indies, we decided to consider both a MAT titer of 400 as a gold standard and a 1/400 dilution of the serum for the Vertical Flow RDT evaluation. It is highly probable that the use of a lower dilution (1/200 or 1/100) would result in both an increased sensitivity and a decreased specificity.

Our results demonstrate that this new rapid diagnostic test could prove useful in endemic contexts, especially in low and middle-income countries. Actually, most of the leptospirosis burden occurs in the back-country with delayed access to the reference laboratories. In epidemics situations, especially during post-disaster periods like in the Philippines in 2009 [45], reference diagnostic tests are seldom if ever available. Therefore, a RDT with good diagnostic performances would also be particularly useful [1]. For easier use, further development of the technique could allow its use with capillary blood.

However, because an early initiation of antibiotherapy is a major contributor to a rapid recovery, the recommendation of treating the patient on the sole basis of a clinical and epidemiological suspicion should be maintained. The use of this Vertical Flow RDT as an initial screen for leptospiral infections would still allow facilitating the difficult differential diagnosis of leptospirosis [46]. Lastly, because the MAT provides important epidemiological information at the population level, it should still be recommended that sera be sent to the reference laboratory for subsequent confirmation by MAT, as suggested by other authors [41].

## Supporting Information

Figure S1 STARD flowchart.
(PDF)

Table S1 MAT Panels used at Institut Pasteur in New Caledonia and at French National Reference Center at Institut Pasteur in Paris.
(DOCX)

Table S2 STARD checklist.
(DOC)
