# Supplementary figures and images for: Sensitivity and Specificity of a New Vertical Flow Rapid Diagnostic Test for the Serodiagnosis of Human Leptospirosis
Source: PLoS Negl Trop Dis. 2013 Jun 27;7(6):e2289. doi: 10.1371/journal.pntd.0002289 (PMC3694835; doi:10.1371/journal.pntd.0002289)

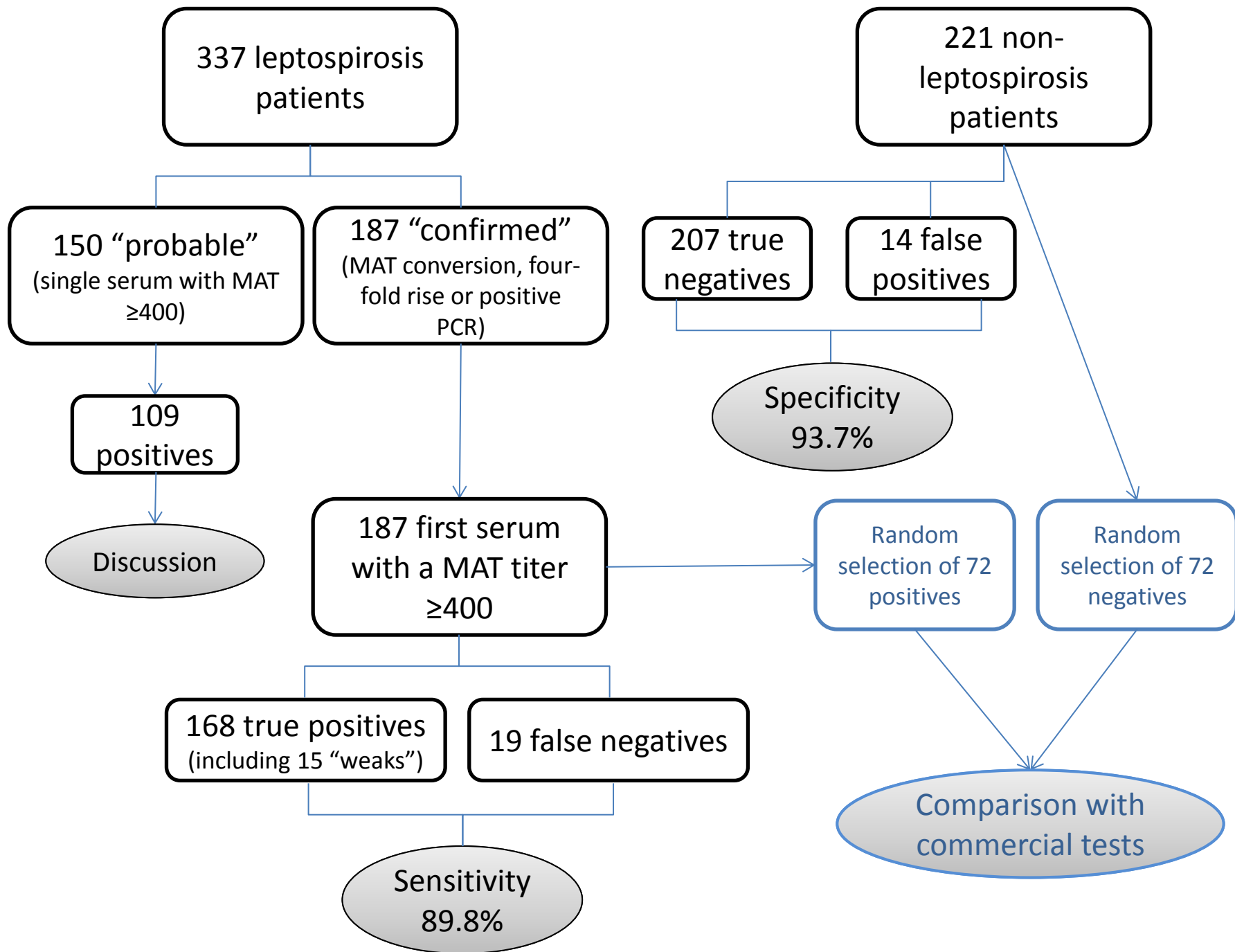

Supplement: Figure S1 — STARD flowchart. (PDF) [file pntd.0002289.s001.pdf]
