# Supplementary material for: Sensitivity and Specificity of a New Vertical Flow Rapid Diagnostic Test for the Serodiagnosis of Human Leptospirosis
Source: PLoS Negl Trop Dis. 2013 Jun 27;7(6):e2289. doi: 10.1371/journal.pntd.0002289 (PMC3694835; doi:10.1371/journal.pntd.0002289)
Supplement: Table S1 — MAT Panels used at Institut Pasteur in New Caledonia and at French National Reference Center at Institut Pasteur in Paris. (DOCX) [file pntd.0002289.s002.docx]

MAT Panel used at Institut Pasteur in New Caledonia

| Serogroup | Serovar | Strain |
| --- | --- | --- |
| Australis | Australis | Ballico |
| Autumnalis | Autumnalis | Akiyami A |
| Ballum | Ballum | Castellon 3 |
| Bataviae | Bataviae | Van Tienen |
| Canicola | Canicola | Hond Utrecht |
| Icterohaemorrhagiae | Icterohaemorrhagiae | Verdun |
| Icterohaemorrhagiae | Copenhagenii | Winjberg |
| Panama | Panama | CZ 214 K |
| Pomona | Pomona | Pomona |
| Pyrogenes | Pyrogenes | Salinem |
| Tarassovi | Tarassovi | Mitis Johnson |
| Semaranga | Patoc | Patoc I |

MAT Panel used at French National Reference Center at Institut Pasteur in Paris

| Serogroup | Serovar | Strain |
| --- | --- | --- |
| Australis | Australis | Ballico |
| Autumnalis | Autumnalis | Akiyami A |
| Ballum | Ballum | Castellon 3 |
| Bataviae | Bataviae | Van Tienen |
| Canicola | Canicola | Hond Utrecht |
| Cynopteri | Cynopteri | 3522 C |
| Grippotyphosa | Grippotyphosa | Moskva V |
| Sejroe | Hardjo | Sponselee |
| Sejroe | Sejroe | M 84 |
| Hebdomadis | Hebdomadis | Hebdomadis |
| Icterohaemorrhagiae | Icterohaemorrhagiae | Verdun |
| Icterohaemorrhagiae | Copenhagenii | Winjberg |
| Panama | Panama | CZ 214 K |
| Pomona | Pomona | Pomona |
| Pyrogenes | Pyrogenes | Salinem |
| Tarassovi | Tarassovi | Mitis Johnson |
| Semaranga | Patoc | Patoc I |
| Celledoni * | Undetermined | 2011/01963 |
| Djasiman * | Djasiman | Djasiman |
| Mini * | Undetermined | 2008/01925 |
| Sarmin * | Sarmin | Sarmin |
| Shermani * | Shermani | 1342 K |
| Javanica * | Javanica | Poi |
| Louisiana * | Louisiana | LUC1945 |

* Serogroups included from January 2011 on
